# Phthalates and type 1 diabetes: is there any link?

**DOI:** 10.1007/s11356-018-1997-z

**Published:** 2018-04-21

**Authors:** Cíntia Castro-Correia, Luísa Correia-Sá, Sónia Norberto, Cristina Delerue-Matos, Valentina Domingues, Cristina Costa-Santos, Manuel Fontoura, Conceição Calhau

**Affiliations:** 1 Unit of Pediatric Endocrinology, Pediatrics Service, São João Integrated Pediatric Hospital, School of Medicine of the University of Porto, Porto, Portugal; 2 Hospital S João, Serviço de Pediatria, Alameda Hernâni Monteiro, 4200 Porto, Portugal; 3 REQUIMTE/LAQV-GRAQ, Institute of Engineering of Porto of the Polytechnic Institute of Porto, Rua Dr. António Bernardino de Almeida, 431, 4200-072 Porto, Portugal; 4 Department of Biochemistry, School of Medicine, University of Porto, Porto, Portugal; 5 MEDCIDS - Department of Community Medicine, Health Information and Decision, School of Medicine, University of Porto, Porto, Portugal; 6 Center for Research in Health Technologies and Information Systems (Centro de Investigação em Tecnologias e Serviços de Saúde – CINTESIS), Porto, Portugal; 7 Nutrition and Metabolism, NOVA Medical School, Universidade Nova de Lisboa, Campo dos Mártires da Pátria, 130, 1169-056 Lisbon, Portugal

**Keywords:** Phthalates, Type 1 diabetes

## Abstract

Phthalates are a group of chemical compounds used as plasticizers in the manufacture of plastic materials. They can be present in many commonly used products. There seems to be a relationship between exposure to phthalates and the occurrence of metabolic dysfunctions, such as a decrease in glucose tolerance, oxidative stress, loss of beta cells, and a decrease in insulin synthesis. As beta cells play a key role in the onset of type 1 diabetes mellitus (T1DM), we sought to investigate the relationship between exposure to phthalates and the diagnosis of T1DM in prepubertal children. Design concentrations of phthalate metabolites were compared in the urine of a population of prepubertal children with new-onset diabetes, patients with T1DM diagnosed more than 6 months previously, and healthy control children. Although the concentrations of DBP and DiBP metabolites were statistically identical in the new-onset diabetes, diabetes, and control groups, there was a clear trend for higher levels of DiBP metabolites in the children with new-onset diabetes. In our sample, there was a trend for higher levels of DiBP metabolites in children with new-onset diabetes.

## Introduction

Phthalates are a group of chemical compounds used in the manufacture of plastic materials, such as PVC (Wittassek et al. 2007). They are frequently used as plasticizers (Koch and Calafat 2009). Since phthalates are not chemically bound to the plastic structure, these compounds may be released to the air and other media with which they are in contact. Their main route of entry into the body is through ingestion of contaminated food (Dong et al. 2017). Because plastic materials containing phthalates as plasticizers are used on a large scale, phthalates can be present in toys, building materials, food packaging, and various other commonly used products (Fourth Report 2009). In recent years, phthalate production has increased significantly (Baillie-Hamilton 2002), and their importance in public health is reflected, for example, by the fact that the majority of the European population excrete phthalate metabolites in their urine samples (Schwedler et al. 2017).

Among phthalates, the compound dibutyl phthalate (DBP) stands out as one of the most abundant components of some plastics (Wittassek et al. 2007).

DBP is not chemically bound to PVC and may, over time, contaminate other materials (Moreira et al. 2013).

Phthalates have the capacity to function as endocrine disruptors. They seem to interfere with a multitude of mechanisms, influencing the hormonal status (Sun et al. 2017).

Phthalates are able to disrupt the hypothalamic-pituitary-thyroid axis (HPTA) (Sun et al. 2017). They seem to play a major role in testicular dysgenesis syndrome, a syndromic complex accounting for a number of genito-reproductive disorders (Skakkebaek et al. 2003).

Phthalates may alter glucose metabolism and adipogenesis (Grun and Blumberg 2007; Casals-Casas et al. 2008; Desvergne et al. 2009). Elevated levels of phthalates have been associated with an increased BMI and waist circumference in adults and children (Stahlhut et al. 2007; Hatch et al. 2008).

Perinatal exposure to DEHP in rats results in important alterations in glucose homeostasis, which are gender- and age-dependent (Lin et al. 2011).

The onset of type 2 diabetes mellitus and metabolic syndrome after exposure to phthalates seems to have insulin resistance as its underlying mechanism (Song et al. 2016), and some studies have demonstrated that alterations in oxidative stress, induced by phthalates, are a causative factor (Kim et al. 2013).

Exposure to phthalates leads to a reduction in the pancreatic insulin content, a loss of beta cells, and an abnormal ultrastructural pattern of these cells (Lin et al. 2011). Human and rat studies have shown that the presence of DEHP is associated with DNA damage (Caldwell 2012). This change may induce an alteration in insulin synthesis. Many studies have been conducted on DEHP, but much less investigation has been carried out on other phthalates, such as DBP and DiBP.

Given that beta cells play a key role in the onset of type 1 diabetes mellitus (T1DM) and that many of the changes described above cause a decrease in the number of these cells, a relationship between the exposure to DBP and DiBP and the diagnosis of T1DM was investigated in a prepubertal children group in this study.

## Materials and methods

### Participants

From a total of 302 T1DM patients followed up at the pediatric endocrinology clinic, 20 patients (12 males and 8 females) were randomly selected based on the assumption that they were prepubertal. These patients were aged 6 to 10 years, had a body mass index (BMI) below the 85th percentile, and were diagnosed with T1DM more than 6 months previously; they had no microvascular complications and no other diseases, and they did not use any associated medications. In the same period of time (2012–2014), all children with an initial diagnosis of diabetes (< 6 months after diagnosis) who visited the Endocrinology Clinic of the São João Hospital (*n* = 8) were included. São João is a university general hospital focused on providing the best health care, with high levels of competence and excellence, and encouraging training and research.

The control group included healthy children followed up at the pediatric surgery unit (*n* = 10) and hospitalized for minor planned surgical procedures.

All the diabetic children were treated with glargine and rapid-acting analogues (lispro, aspart, or glulisine) through multiple daily injections, according to the ISPAD (International Society of Pediatric and Adolescent Diabetes) guidelines (Danne et al. 2014).

All urine samples were collected in glass containers so that the contamination factor was reduced.

The project was approved by the Institutional Ethics Committee, and all patients’ parents/guardians signed an informed consent form prior to the commencement of the study.

### Chemical analysis

Urine samples were collected on the same day and stored at − 20 °C until analysis. The samples were analyzed for phthalates and metabolites via solid-phase extraction-gas chromatography/mass spectrometry (SPE-GC/MS) after enzymatic hydrolysis with beta-glucuronidase overnight at 37 °C (from *E. coli*, without arylsulfatase activity); the internal standard DEHP-3,4,5,6-d4 was also added. Elution was performed with ethyl acetate, and then the samples were dried under a gentle stream of N_2_ and redissolved in 100 μL of the derivatization reagent (N,O-bis(trimethylsilyl)trifluoroacetamide (BSTFA) + 1% trimethylchlorosilane (TMCS)). This mixture was placed in a water bath at 80 °C for 30 min. The final extract was transferred to a glass insert (in a GC vial) and analyzed using the GC-MS system. Chromatographic analyses were carried out in a TRACE GC Ultra gas chromatograph with Polaris Q coupled to an ion trap mass spectrometer (Thermo Fisher Scientific) operated in electron impact ionization (EI) mode at 70 eV and controlled by Xcalibur 1.3. The helium carrier gas (Linde Sógas, purity ≥ 99.999%) was maintained at a constant flow of 1 mL/min. Injections (2 μL) were carried out in splitless mode. A Phenomenex ZB-XLB column (30 m × 0.25 mm I.D., 0.25-μm film thickness) was used. The GC oven temperature was programmed from an initial temperature of 90 °C (1 min hold), ramped up at 15 °C/min to 250 °C (1 min hold), increased to 255 °C at 20 °C/min (5 min hold), and finally increased to 270 °C at 10 °C/min (1 min hold). This program resulted in a total run time of 23.42 min. The other optimized parameters included a transfer line temperature of 270 °C and an ion source temperature of 250 °C. The compounds were quantified with selected ion monitoring (SIM). Identification was confirmed by the retention times and ion ratios.

The method detection limits (MDLs) in the urine were 0.0011 and 0.00018 mcg/L for mono-isobutyl phthalate (MiBP) and monobutyl phthalate (MBP), respectively.

### Statistical methods

Some of the evaluated children presented with values lower than the limit of detection (LOD). Therefore, the groups (diabetes, new diabetes and control) were compared using Fisher’s exact test in terms of the percentage of children who had measurable levels of phthalate metabolites in their urine.

The Kruskal-Wallis test was then used to compare phthalate levels among the groups in only those cases in which these levels were measurable.

A significance level of 0.05 was used for all tests.

## Results

The children were divided into three groups as follows: without diabetes (controls); with T1DM for more than 6 months (diabetes); and new-onset diabetes (new diabetes).

The characteristics of the patients are described in Table 1.

**Table 1:** Characteristics of the evaluated patients by groups

	Controls (*n* = 10) median (min–max)	Diabetes (*n* = 20) median (min–max)	New diabetes (*n* = 8) median (min–max)
Age at diagnosis (years)	–	3.2 (0.9–9.1)	8.9 (2.3–9.7)
Current age (years)	5.3 (3.1–8.6)	7.3 (3.5–11.3)	9.0 (2.2–9.7)
Illness duration (years)	–	2.6 (0.3–7.2)	–
BMI	17.0 (15.4–20.8)	17.0 (15.8–22.1)	16 (14.5–19.2)
HbA1c (%)	–	8.3 (6.6–10.2)	9.4 (8.2–10.6)

Some of the children evaluated had phthalate concentrations lower than the LOD, and others had phthalate concentrations lower than the LOQ. Neither of these groups were taken into account. No statistically significant differences were found among the groups in the percentage of cases with measurable levels of phthalates (Table 2).

**Table 2:** Statistics of the cases who had measurable levels of phthalates

	Controls (*n* = 10) *n* (%)	Diabetes (*n* = 20) *n* (%)	New diabetes (*n* = 8) *n* (%)	*p*
MIBP	9 (90)	15 (75)	7 (88)	0.640
MBP	2 (20)	3 (15)	3 (38)	0.435

MiBP is a urinary metabolite of the parental chemical diisobutyl phthalate (DiBP), and MBP is a di-n-butyl phthalate (DBP) metabolite.

Considering only the children who had detectable phthalate concentrations in the urine, there were no statistically significant differences in the DBP and DiBP urine metabolites among the groups (Table 3). Nevertheless, there was a clear trend towards higher median values in the children with inaugural diabetes.

**Table 3:** Median (minimum–maximum) values for the patients who had measurable phthalate levels

	Controls mcg/l	Diabetes mcg/l	New Diabetes mcg/l	*p*
MIBP	8.4 (1.4–24.9)	11.8 (0.2–229.8)	40.3 (6.7–101.2)	0.117
MBP	29.1 (1.1–57)	13.8 (5.2–151)	39.3 (29.9–851.9)	0.566

## Discussion

For many reasons, children are more susceptible to the harmful effects of endocrine disruptors. A child’s metabolic response to the presence of these substances is different from that of adults (Ginsberg et al. 2004; Faustman et al. 2000). Children have higher metabolic needs, with a greater need for food and water intake per body weight (Moya et al. 2004). Children are also often more exposed to phtalate-containing materials because of physiological and behavioral factors. They may have behaviors that pose a greater risk of contact with contaminants, such as placing objects in their mouths and crawling (Moya et al. 2004), especially younger children.

Diisobutyl phthalate (DIBP) is a colorless, transparent, oily liquid used as an alternative to DBP (dibutyl phthalate). It is used in nitrocellulose and alkyd resin paints. Both DIBP and DBP are plasticizers. Phthalates are not tightly bound to the plastic; therefore, they are easily released into the environment, accumulating in the dust in a house (Ginsberg et al. 2016). Both plasticizers are found to be major contributors to phthalate exposure through ingestion (Weldingh et al. 2017).

Exposure to these substances has been decreasing in the past few years, likely due to the legislation regarding their use. However, despite the existence of European or American laws, there are other countries that do not follow the same rules and still use plastics with a higher percentage of phthalates, including in toys.

In Portugal, the presence of some phthalates in tap water, as well as in several samples of bottled water (mostly in plastic bottles), was investigated (Santana et al. 2014). Three phthalates (dibutyl (DnBP), diisobutyl (DIBP) and DEHP) were found (Santana et al. 2014). The concentration of DEHP was approximately five times higher in the water in plastic bottles than in the water in glass bottles (Santana et al. 2014). In tap water, only DIBP and DEHP were found (Santana et al. 2014). All samples had phthalate concentrations below 6 μg/l, which is the maximum allowed concentration in water established by the US Environmental Protection Agency (Santana et al. 2014).

Samples from children in our study contained MiBP, a DiBP metabolite, with a much smaller percentage of samples containing DBP metabolites.

Phthalates are substances that function as endocrine disruptors. They may show insulin resistance-inducing effects and aggravate metabolic syndrome (Grun and Blumberg 2007). There is also evidence of some direct effects on beta cells, with a consequent decrease in their insulin secretory potential (Tordjman et al. 2002).

In the past few decades, the incidence of diabetes has increased markedly worldwide (Weldingh et al. 2017). This increase coincides with a marked increase in exposure to a number of synthetic chemicals, including several substances known to have the capacity for endocrine disruption (WHO 2013).

These facts led to the concept of a possible relationship between the exposure to endocrine disruptors and the increase in diabetes mellitus (Neel and Sargis 2011).

T1DM is an autoimmune disease characterized by an extensive loss of pancreatic beta cells and consequent insulin deficiency and hyperglycaemia (Mathis et al. 2001). The underlying mechanism is a process of cell death, which is induced by cytokines (Pirot et al. 2008).

Although several studies have sought to establish a relationship between phthalate exposure and the onset of type 2 diabetes, the relationship between these substances and T1DM has been investigated less frequently (Weldingh et al. 2017).

Lin et al. have shown that in rats, exposure to DEHP during pregnancy led to changes in glucose metabolism in the offspring (Lin et al. 2011). Additionally, direct exposure of pancreatic cells to DEHP caused apoptosis and altered insulin secretion (Sun et al. 2015).

The development of pancreatic autoimmunity precedes the destruction of beta cells and the diagnosis of T1DM (Eisenbarth 1986). Studies have shown a possible relationship between the presence of DBP and the development of immunity (Lourenço et al. 2015). Phthalates, namely, DBP, seem to be able to induce thyroiditis, also an autoimmune disease (Wu et al. 2017).

We evaluated the possibility of the same phenomenon with regard to beta cells, with possible autoimmune mechanisms triggered by the exposure to these endocrine disruptors.

Although the median values in the diabetes and new diabetes groups of children were higher than those in the controls, there were no statistically significant differences among the three groups. However, there was a clear trend for the children with new-onset diabetes to have higher urinary concentrations of phthalate metabolites. Certainly, one of the limitations of our investigation is a small sample size, which may have contributed to the lack of statistical significance.

One factor to be taken into account is that all overweight and obese children were excluded because obesity and overweight could be a confounding factor in this analysis.

## Conclusion

No statistically significant differences were found in the concentrations of urinary metabolites of DiBP and DBP among newly diagnosed diabetic children, children with established type 1 diabetes and healthy controls. However, children recently diagnosed with diabetes showed a tendency to have higher DiBP metabolite concentrations. Considering recent data on these pollutants, it is imperative to conduct clinical studies with larger population samples.

## Abbreviations

BMI: Body mass index
DEHP: Di(2-ethylhexyl)phthalate
DBP: Dibutyl phthalate
DIBP: Diisobutyl phthalate
LOD: Limit of detection
MBP: Monobutyl phthalate
MiBP: Mono-isobutyl phthalate
T1DM: Type 1 diabetes mellitus

## References

[CR1] Baillie-Hamilton PF (2002). Chemical toxins: a hypothesis to explain the global obesity epidemic. J Altern Complement Med.

[CR2] Caldwell JC (2012). DEHP: genotoxicity and potential carcinogenic mechanisms—a review. Mutat Res.

[CR3] Casals-Casas C, Feige JN, Desvergne B (2008). Interference of pollutants with PPARs: endocrine disruption meets metabolism. Int J Obes.

[CR4] Danne T, Bangstad H-J, Deeb L, Jarosz-Chobot P, Mungaie L, Saboo B, Urakami T, Battelino T, Hanas R (2014). Insulin treatment. Pediatr Diabetes.

[CR5] Desvergne B, Feige JN, Casals-Casas C (2009). PPAR-mediated activity of phthalates: a link to the obesity epidemic?. Mol Cell Endocrinol.

[CR6] Dong RH, Zhang H, Zhang MR, Chen JS, Wu M, Li SG, Chen B (2017). Association between phthalate exposure and the use of plastic containers in Shanghai. Biomed Environ Sci.

[CR7] Eisenbarth GS (1986). Type I diabetes mellitus. A chronic autoimmune disease. N Engl JMed.

[CR8] Faustman EM, Silbernagel SM, Fenske RA, Burbacher TM, Ponce RA (2000). Mechanisms underlying Children’s susceptibility to environmental toxicants. Environ Health Perspect.

[CR9] Fourth Report (2009) Fourth Report on human exposure to environmental chemicals. Atlanta, GA: U.S. Department of Health and human services, Centers for disease control and prevention. https://www.cdc.gov/exposurereport/

[CR10] Ginsberg G, Hattis D, Sonawane B (2004). Incorporating pharmacokinetic differences between children and adults in assessing children’s risks to environmental toxicants. Toxicol Appl Pharmacol.

[CR11] Ginsberg G, Ginsberg J, Foos B (2016). Approaches to Children’s exposure assessment: case study with diethylhexylphthalate (DEHP). Int J Environ Res Public Health.

[CR12] Grun F, Blumberg B (2007). Perturbed nuclear receptor signaling by environmental obesogens as emerging factors in the obesity crisis. Rev Endocr Metab Disord.

[CR13] Hatch EE, Nelson JW, Qureshi MM, Weinberg J, Moore LL, Singer M, Webster TF (2008). Association of urinary phthalate metabolite concentrations with body mass index and waist circumference: a cross-sectional study of NHANES data, 1999–2002. Environ Health.

[CR14] Kim JH, Park HY, Bae S, Lim YH, Hong YC (2013). Diethy lhexyl phthalates is associated with insulin resistance via oxidative stress in the elderly: a panel study. PLoS One.

[CR15] Koch HM, Calafat AM (2009). Human body burdens of chemicals used in plastic manufacture. Philos Trans R Soc Lond B Biol Sci.

[CR16] Lin Y, Wei J, Li Y, Chen J, Zhou Z, Song L, Wei Z, Lv Z, Chen X, Xia W, Xu S (2011). Developmental exposure to di(2-ethylhexyl) phthalate impairs endocrine pancreas and leads to long-term adverse effects on glucose homeostasis in the rat. Am J Physiol Endocrinol Metab.

[CR17] Lourenço AC, Galbiati V, Corti D, Papale A, Martino-Andrade AJ, Corsini E (2015). The plasticizer dibutyl phthalate (DBP) potentiates chemical allergen-induced THP-1 activation. Toxxicol in Vitro.

[CR18] Mathis D, Vence L, Benoist C (2001). β-cell death during progression to diabetes. Nature.

[CR19] Moreira MA, André LC, Cardeal ZL (2013). Analysis of phthalate migration to food simulants in plastic containers during microwave operations. Int J Environ Res Public Health.

[CR20] Moya J, Bearer CF, Etzel RA (2004). Children’s behavior and physiology and how it affects exposure to environmental contaminants. Pediatrics.

[CR21] Neel BA, Sargis RM (2011). The paradox of progress: environmental disruption of metabolism and the diabetes epidemic. Diabetes.

[CR22] Pirot P, Cardozo AK, Eizirik DL (2008). Mediators and mechanisms of pancreatic beta-cell death in type 1 diabetes. Arq Bras Endocrinol Metabol.

[CR23] Santana J, Giraudi C, Marengo E, Robotti E, Pires S, Nunes I, Gaspar EM (2014). Preliminary toxicological assessment of phthalate esters from drinking water consumed in Portugal. Environ Sci Pollut Res Int.

[CR24] Schwedler G, Seiwert M, Fiddicke U, Ißleb S, Hölzer J, Nendza J, Wilhelm M, Wittsiepe J, Koch HM, Schindler BK, Göen T, Hildebrand J, Joas R, Joas A, Casteleyn L, Angerer J, Castano A, Esteban M, Schoeters G, Den Hond E, Sepai O, Exley K, Bloemen L, Knudsen LE, Kolossa-Gehring M (2017). Human biomonitoring pilot study DEMOCOPHES in Germany: Contribution to a harmonized European approach. Int J Hyg Environ Health.

[CR25] Skakkebaek NE, Holm M, Hoei-Hansen C, Jørgensen N, Rajpert-De ME (2003). Association between testicular dysgenesis syndrome (TDS) and testicular neoplasia: evidence from20 adult patients with signs of maldevelopment of the testis. Acta Pathol. Microbiol. Immunol. Scand.

[CR26] Song Y, Chou EL, Baecker A, You NC, Song Y, Sun Q, Liu S (2016). Endocrine-disrupting chemicals, risk of type 2 diabetes, and diabetes-related metabolic traits: a systematic review and meta-analysis. J Diabetes Jul.

[CR27] Stahlhut RW, van Wijngaarden E, Dye TD, Cook S, Swan SH (2007). Concentrations of urinary phthalate metabolites are associated with increased waist circumference and insulin resistance in adult U.S. males. Environ Health Perspect.

[CR28] Sun Y, Lin Q, Huang Q, Shi J, Qiu L, Kang M (2015). Di(2-ethylhexyl) phthalate-induced apoptosis in rat INS-1 cells is dependent on activation of endoplasmic reticulum stress and suppression of antioxidant protection. J Cell Mol Med.

[CR29] Sun D, Zhou L, Wang S, Liu T, Zhu J, Jia Y, Xu J, Chen H, Wang Q, Xu F, Zhang Y, Ye L (2017). Effect of Di-(2-ethylhexyl) phthalate on the hypothalamus-pituitary-thyroid axis in adolescent rat. Endocr J.

[CR30] Tordjman K, Standley KN, Bernal-Mizrachi C, Leone TC, Coleman T, Kelly DP, Semenkovich CF (2002). PPARalpha suppresses insulin secretion and induces UCP2 in insulinoma cells. J Lipid Res.

[CR31] Weldingh Nina Mickelson, Jørgensen-Kaur Lena, Becher Rune, Holme Jørn A., Bodin Johanna, Nygaard Unni C., Bølling Anette Kocbach (2017). Bisphenol A Is More Potent than Phthalate Metabolites in Reducing Pancreatic β-Cell Function. BioMed Research International.

[CR32] Wittassek M, Heger W, Koch HM, Becker K, Angerer J, Kolossa-Gehring M (2007). Daily intake of di (2-ethylhexyl) phthalate (DEHP) by German children—a comparison of two estimation models based on urinary DEHP metabolite levels. Int J Hyg Environ Health.

[CR33] World Health Organization (2013) Fact sheet diabetes: global report on diabetes, http://www.who.int/diabetes/global-report/en/. Global Industry Analysts, “Bisphenol A—A Global Strategic Business Report”

[CR34] Wu Y, Li J, Yan B, Zhu Y, Liu X, Chen M, Li D, Lee CC, Yang X, Ma P (2017). Oral exposure to dibutyl phthalate exacerbates chronic lymphocytic thyroiditis through oxidative stress in female Wistar rats. Sci Rep.

